# RNA-Seq Revealed Novel Non-proliferative Retinopathy Specific Circulating MiRNAs in T2DM Patients

**DOI:** 10.3389/fgene.2019.00531

**Published:** 2019-06-04

**Authors:** Zimeng Li, Ying Dong, Chang He, Xingchen Pan, Dianyuan Liu, Jianli Yang, Liankun Sun, Peng Chen, Qing Wang

**Affiliations:** ^1^Department of Endocrinology, China-Japan Union Hospital, Jilin University, Changchun, China; ^2^Department of Radiotherapy, The Tumor Hospital of Jilin Province, Changchun, China; ^3^Department of Genetics, College of Basic Medical Sciences, Jilin University, Changchun, China; ^4^Department of Molecular Biology, College of Basic Medical Sciences, Jilin University, Changchun, China; ^5^Department of Pathophysiology, College of Basic Medical Sciences, Jilin University, Changchun, China; ^6^Department of Pathology, College of Basic Medical Sciences, Jilin University, Changchun, China

**Keywords:** diabetes retinopathy, type 2 diabete mellitus, microRNA, polygenic risk score, early prediction

## Abstract

**Background:** Diabetic retinopathy (DR) is a common diabetes complication and was considered as the major cause of blindness among young adults. MiRNAs are a group of small non-coding RNAs regulating the expression of target genes and have been reported to be associated with the development of DR in a variety of molecular mechanisms. In this study, we aimed to identify miRNAs that are differentially expressed (DE) in the serum of DR patients.

**Methods:** We recruited 21 type 2 diabetes mellitus (T2DM) inpatients of Chinese Han ancestry, consisting of 10 non-proliferative DR patients (DR group) and 11 non-DR T2DM patients (NDR group). MiRNA was extracted from fasting peripheral serum and quantified by RNA-seq. The expression levels of miRNA were evaluated and compared between the two groups, with adjustments made for age differences. The validated target genes of miRNAs were subjected to a pathway analysis. We also constructed a weighted polygenic risk score using the DE miRNA and evaluated its predictive power.

**Results:** Five miRNAs were DE between DR and NDR groups (*p*-Value ≤ 0.01, LFC ≥ 2 or LFC ≤-2). These included miR-4448, miR-338-3p, miR-190a-5p, miR-485-5p, and miR-9-5p. In total, these miRNAs were validated to regulate 55 target genes. Four target genes were found to overlap with the NAD metabolism, sirtuin, and aging pathway, which was thought to control the vascular growth and morphogenesis. The predictive power of our polygenic risk score was apparently high (AUC = 0.909). However, it needs to be interpreted with caution.

**Conclusion:** In this study, we discovered novel DR-specific miRNAs in human serum samples. These circulating miRNAs may represent the pathological changes in the retina in response to diabetes and may serve as non-invasive biomarkers for early DR risk prediction.

## Introduction

Diabetic retinopathy (DR) is one of the most common and serious complications in diabetes and considered as the leading cause of blindness in adults (Zhang et al., 2018). Moreover, people with DR are at high risk of other micro- and macrovascular complications from diabetes (Joglekar et al., 2016). The etiology of DR have been extensively studied, however, the mechanisms are still unclear. Until now, DR can only be diagnosed through retinal examination (Shaker et al., 2018) and the prognosis of almost all treatment remains poor (Stitt et al., 2016). Furthermore, the effectiveness of biomarkers for early detection needs to be improved (Ye Z. et al., 2017; Liang et al., 2018).

MiRNAs are a group of small non-coding RNAs with a length of 19–24 nucleotides, which are capable of regulating the expression of specific genes (Ye Z. et al., 2017). MiRNAs have been noted to be very stable in a variety of body fluids, such as serum, plasma, saliva, tears, aqueous and vitreous humor, and urine (Ye and Steinle, 2017; Shaker et al., 2018). In addition to their regulatory functions of gene expression and as a potential therapeutic target, miRNA is considered as a useful and easily accessible diagnostic marker for many diseases, including non-small-cell lung cancer (Shao et al., 2017), breast cancer (Li et al., 2016), gastric cancer (Pereira et al., 2019), coronary artery disease (Zhang et al., 2019), as well as a potential mediator of physiological and pathological processes (Ye and Steinle, 2017). They can be released from their cells of origin and circulate to different target cells in different tissues (Elmasry et al., 2018). Thus, peripheral blood circulating miRNAs have emerged as both novel biomarkers of diabetes and therapeutic targets for disease treatment (Gong and Su, 2017; Kamalden et al., 2017; Ye E.A. et al., 2017; Pastukh et al., 2019).

Although the diagnostic and therapeutic potential of circulating miRNAs was particularly profound in cancers, there were evidences showing that dozens of circulating miRNAs were dysregulated in type 1 and type 2 diabetes (Terrinoni et al., 2018). As for clinical applications, therapies which target pathogenic miRNAs have entered clinical trials, including tumor suppressor miR-34 and hepatitis related miR-122 (Kalariya et al., 2017; Rupaimoole and Slack, 2017).

Until now, there has been limited understanding of the expression profile of total miRNA in circulating serum of DR patients. Our goal in this study is to identify miRNAs that are differentially expressed (DE) in DR patients, and further investigate whether circulating miRNAs can be used as biomarkers in predicting the DR onset in type 2 diabetes mellitus (T2DM) patients. We think that our results would be valuable to the clinical management and mechanism studies of microvascular complications of T2DM.

## Materials and Methods

### Study Subjects

T2DM patients from inpatients of the endocrinology department of China-Japan Union Hospital of Jilin University were recruited between November 2016 and May 2017. All the patients were subjected to a detailed medical examination and retinopathy diagnosis. The T2DM diagnoses were made *as per* the diagnostic criteria and classification of diabetes of World Health Organization (1999). In brief, people were classified as T2DM patients if they showed diabetes symptoms (e.g., feeling very thirsty, urinating often, and losing weight) and met one of the following criteria: fasting blood glucose ≥ 7.0 mmol/L, 2-h post 75 g glucose load blood glucose ≥ 11.1 mmol/L or random blood glucose ≥ 11.1 mmol/L. For those who were not showing diabetes symptoms, a second blood glucose test was performed to confirm the diagnosis. All these patients were negative for Type 1 diabetes antibodies.

According to the international clinical classification systems for diabetic retinopathy in the fundus disease academic conference in 2002 (Wilkinson et al., 2003), T2DM patients were divided into two groups. The DR group consisted of T2DM patients with less than 5 years of diabetes duration (*N* = 10), while the NDR group was consisted of T2DM patients with more than 10 years of diabetes duration (*N* = 11). The DR diagnosis was not made until at least 2 physicians reached a consistent conclusion. We excluded patients with diabetic complications other than retinopathy, including nephropathy, peripheral neuropathy, ketoacidosis, hyperosmolar coma, and other acute complications of diabetes. Patients with other major diseases, such as severe stress (e.g., recent cardiovascular events or trauma surgery), acute or chronic infection, liver diseases, rheumatic diseases or tumor, were also excluded.

This study was approved by China-Japan Union Hospital of Jilin University and complied with the guidelines and principles of the Declaration of Helsinki. Written informed consent was obtained from all the participants or their legal representatives.

### Data Collection

All the patients were subjected to a detailed questionnaire, medical examinations, laboratory testing and fundus ophthalmoscope examination by using a non-mydriasis retinal camera (Canon, Tokyo, Japan). The height and weight were measured and used to calculate the body mass index (BMI, kg/m^2^). Other medical information, e.g., age, gender, self and family disease history, diabetes course, glycemic control, medication history, allergic history, and smoking and drinking behaviors, were collected using a questionnaire.

### Serum Collection and Storage

The selected subjects fasted for 8–12 h. Approximately 5 ml of venous blood sample was extracted in the early morning in a citrate anticoagulant tube at room temperature. The venous whole blood samples were centrifuged at 1000*g* for 10 min. The serum was taken and stored at -80°C for subsequent use.

### RNA-Seq

Serum samples were shipped to the sequencing company on dry ice (Kangchen, Shanghai, China). Total RNA was extracted from serum samples using Trizol (Invitrogen, Wisconsin, United States). Sequencing libraries were prepared using NEBNext small RNA library prep set for Illumina (New England Biolabs). The PCR (polymerase chain reaction) products of about 135–155 bp, corresponding to small RNAs of 15–35 nt in length, were gel purified and sequenced on an Illumina NextSeq 500 sequencer.

### MiRNA Expression Quantification

We adopted miRDeep2 (version 0.0.8) pipeline to identify and quantify expressed miRNAs in our samples. Briefly, the adapter sequences were clipped from raw reads. The clipped read of length 18 nt and above were aligned to reference genome (build GRCh38) by bowtie (version 1.2.1). Finally, the core miRDeep2 algorithm was invoked to quantify the read counts which have been mapped to miRNA hairpins and mature form (miRBase release 21).

### Statistical Analysis and Differential Expression Analysis

All statistical analysis of clinical data was done using SPSS 21.0 software (IBM, NY, United States). The differences between groups were compared with *t-*test for age and BMI, or fisher’s exact test for sex. A *p*-value of 0.05 or less was considered as a significant difference in sex, age and BMI.

Read counts mapped to the same mature miRNA, derived from different precursors, were merged together. The differential expression of miRNA between DR and NDR group was estimated in a general linear model (DESeq2, version 1.22.1), adjusted for age. The raw count data from RNA-seq were modeled under a negative binomial distribution. The logarithm of the normalized fragments per million (FPM) was calculated and used to estimate the coefficients of the general linear model using Wald test. MiRNAs of insufficient abundance (total count < 200) were excluded from the analysis. The *p*-values were corrected for multiple testing using the procedure proposed by Benjamini and Hochberg (1995).

### Target Genes of Differentially Expressed MiRNAs and Pathway Analysis

The target genes regulated by miRNAs can be either predicted using algorithms or validated using biological experiments. In this study, we investigated the target genes that have been validated using luciferase reporter assay to be regulated by one of the DE miRNAs (Ru et al., 2014). We employed FUMA gene2func online tool to reveal the pathways defined in WikiPathways that the target genes have a role in Watanabe et al. (2017); Slenter et al. (2018).

### Polygenic Risk Score and Prediction Power of DR in T2DM Patients

In the scenario of single miRNA, the DR risk of a T2DM patient was estimated using the logarithm of normalized FPM of the miRNA *per se*. For the polygenic risk score constructed with multiple miRNAs, the logarithms of the normalized FPM across multiple miRNAs were summed together, weighted by the log2 fold change (LFC) of the miRNAs, respectively. The area under the curve (AUC), sensitivity and specificity, and optimal cutoff were estimated using the pROC package of R (https://www.r-project.org).

## Results

### Characteristics of DR and NDR Groups

Twenty-one T2DM patients were included in our study. All samples were of Chinese Han ethnicity. DR group has four cases of males and six cases of females, with an average BMI of 27.17 ± 3.79. NDR group has seven cases of males and four cases of females, with an average age of 25.23 ± 3.03 (**Table 1**). There is no significant difference of sex and BMI between the two groups (*p*-value > 0.05). In order to maximize our capacity of identifying genuine DE miRNA, we intendedly selected DR patients of shorter diabetes duration and NDR group of longer diabetes duration. Therefore, the average age of DR group is 16.35 years younger than that of NDR group (*p* < 0.001). This extreme case-control study design can better surrogate the susceptibility to DR. When the blood samples were taken, the DR patients were all diagnosed as non-proliferative retinopathy by an ophthalmologist.

**Table 1:** Characteristics of DR and NDR groups.

	DR group	NDR group	*P*-value
Number of samples	10	11	n.a
T2DM Course (year)	<5 years	>10 years	n.a
Gender (M/F)	4/6	7/4	0.395
Age (year)	47.10 ± 6.45	63.45 ± 4.25	<0.001
BMI (kg/m^2^)	27.17 ± 3.79	25.23 ± 3.03	0.215


*Data is expressed as mean ± SD. Disease course was recorded according to the questionnaires completed by the patients*.

### Differential MiRNA Expression Between DR and NDR Samples

**FIGURE 1:**
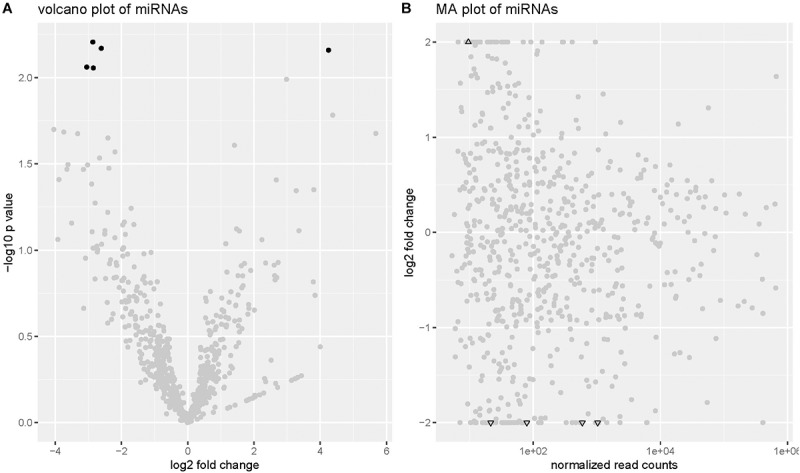
The volcano plot and MA-plot of expressed miRNAs. **(A)** Volcano plot. Significantly DE miRNAs are in black. Others are in gray. **(B)** MA plot. When the LFC of the DE miRNAs were greater than 2 or less than –2, they were presented by black triangles on the boundary.

Sequence data analysis using mirDeep2 revealed 2588 mature miRNAs which were expressed in the 21 serum samples. After restricting our analysis in those with sufficient abundance (total count ≥ 200), there were 756 miRNAs left for differential expression analysis. No significant differential expression was identified after multiple testing corrections. In this study, we regarded miRNAs with *p*-value ≤ 0.01 and LFC ≥ 2 or ≤-2 as significantly differentially expressed. At this significance level, five miRNAs were DE (**Figure 1**). Among these, four miRNAs (miR-4448, miR-338-3p, miR-485-5p, and miR-9-5p) were down-regulated and the remaining (miR-190a-5p) was up-regulated in serum samples of DR patients (**Table 2**).

**Table 2:** Differentially expressed miRNAs in DR serum samples.

Mature miRNA ID	LFC	LFC.SE	*p*-Value
miR-4448	-2.86	1.05	0.006
miR-338-3p	-2.60	0.96	0.007
miR-190a-5p	4.26	1.58	0.007
miR-485-5p	-3.04	1.16	0.009
miR-9-5p	-2.84	1.09	0.009


*Differentially expressed miRNAs with p-Value less than 0.01 and LFC ≥ 2 or LFC ≤-2 were present. Fold change indicates the miRNA level of DR group over NDR group. The p-Values were adjusted for age, without multiple testing correction. LFC, log_2_ fold change; LFC.SE, standard error of log_2_ fold change*.

### Target Genes of the DE MiRNAs and Enriched Pathway

We identified the target genes that have been proved to be regulated by DE miRNAs using luciferase assay. In total, 55 genes were regulated by these miRNAs. Among these, 41 were targeted by miR-9-5p alone (Supplementary Table 1). Pathway analysis revealed that 4 target genes (*SIRT1*, *FOXO1*, *FOXO3*, and *NFKB1*) of miR-9-5p belong to NAD metabolism, sirtuins and aging pathway. There are 11 known genes in this pathway, which were mostly regulated by miR-9-5p (enrichment *p*-value = 1.76 × 10^-9^, corrected *p*-value = 7.13 × 10^-7^). In addition, these 4 target genes were possibly under joint regulation by the DE miRNAs. According to the target gene predictions, we found that *SIRT1* was the potential target of miR-338 and miR-9-5p, while *FOXO1* and *FOXO3* were targeted by miR-485-5p, miR-338-3p, and miR-9-5p at the same time.

### The Performance of MiRNA Polygenic Risk Score

**FIGURE 2:**
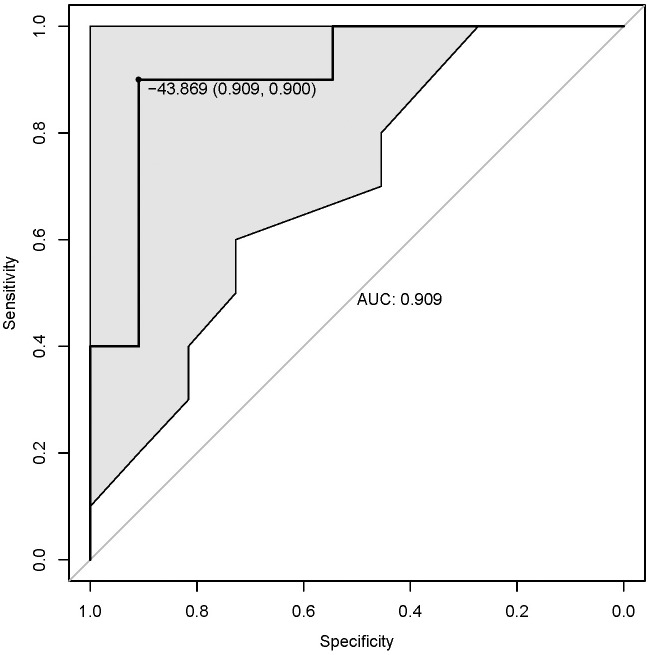
The ROC curve of the polygenic risk score. ROC, receiver operator characteristics; AUC, area under curve. Shadow area represents the 95% confidence interval. The dot in the curve represents the optimal cutoff point and the corresponding specificity and sensitivity (parentheses).

We evaluated the predictive power of individual miRNA with receiver operator characteristics (ROC) curve (Supplementary Figure 1). The AUC values of the 5 DE miRNAs were greater than 0.7, with the highest predictive power from miR-4448 and miR-9-5p (AUC = 0.836). The polygenic score with all five miRNAs was significantly higher in DR samples (*p*-value = 6.3 × 10^-3^, Supplementary Figure 2). The AUC value of the polygenic risk score was estimated as 0.909, with the optimal cutoff -43.869 (specificity = 0.909, sensitivity = 0.900, **Figure 2**).

## Discussion

In this study, we leveraged the high throughput sequencing technology and investigated the DR specific circulating miRNA profile that could be of important relevance in the etiology of DR. Although the sample size of our study was small, we were able to discover the substantially DE (LFC > 2 or LFC < -2) miRNAs under an extreme case-control study design. In fact, we demonstrated that the fold change of DE circulating miRNA levels between the two groups ranged from 6.1 to 19.1. To our knowledge, these miRNAs have never been associated with DR in T2DM patients before this study. The NDR group in this study has a much longer diabetes duration than the DR group, possibly resulting from a different vulnerability to retinopathy under T2DM physiological condition.

The predictive power of these miRNAs seemed to be clinically actionable (AUC = 0.909). However, it is worth noting that the evaluation of AUC was done in the discovery samples, which may inflate the estimated AUC. A validation in an independent and larger cohort is necessary to assess the predictive power of our polygenic score. In addition, the discovery sample size was small and may not represent the general T2DM population well. People should expect a significant drop of predictive power when used in clinical practice. Furthermore, several of T2DM patients were treated with metformin. It has been pointed out that the circulating levels of miR-146a were higher in T2DM patients treated with metformin compared to those with other anti-diabetic medicines (Mensa et al., 2019). Theoretically, our differential expression analysis should take anti-diabetic medication into consideration. However, since our samples came from inpatients, changes in medication after enrollment were frequently seen as part of our clinical practices (data not shown). This made the adjustment of anti-diabetic medicine unpractical.

Retinopathy is a chronic eye disease with two progressive stages, non-proliferative retinopathy and proliferative retinopathy. In non-proliferative DR, high glucose condition was said to induce the dysfunction of blood-retina barrier (BRB) and contributes to the progression of DR (Giebel et al., 2005). Non-proliferative DR may develop into proliferative diabetic retinopathy (PDR) at the advanced stage, which could be characterized by ischemia-induced neovascularization along the retina and the vitreous surface (Aiello et al., 1994). The DR patients included in this study were all classified as non-proliferative DR. Thus, our results indicated the biomarkers of early stage DR and could potentially become predictive markers of DR onset, but were not necessarily capable of estimating the progression of DR.

We also observed substantial overlapping of the target genes of these DR specific miRNAs with the NAD metabolism, sirtuins and aging pathway (Supplementary Figure 3), which is capable of regulating vascular network formation and morphogenesis. Sirtuins are NAD-dependent deacetylases. In particular, SIRT1 was found to be a vital regulator of revascularization in ischemic tissues. With the absence of SIRT1, the blood vessel network formation was impaired (Potente et al., 2007). In fact, SIRT1 deacetylates and destabilize Notch1 intracellular domain in endothelial cells (EC). This can attenuate Notch signaling and negatively regulate its activity (Guarani et al., 2011). Notch signaling controls the important steps of blood vessel formation, promoting stalk behavior but limiting explorative tip or sprout behavior of EC (Phng and Gerhardt, 2009). This explained the phenomenon that zebrafish with *sirt1* knockdown showed sparse blood vessel network (Potente et al., 2007). Moreover, FOXO1 and FOXO3, in collaboration, regulate vascular morphogenesis by repressing sprout and migration of EC (Potente et al., 2005). Finally, FOXOs can be deacetylated by SIRT1 and be further degraded by proteasome. In our study, we found that miR-9-5p was down-regulated in DR samples, possibly indicating a higher level of transcription of both *SIRT1* and *FOXOs*, which play counteracting roles in revascularization. Hence, we speculate that SIRT1 was in epistasis of FOXOs by deacetylation after translation, so that only the effect of SIRT1 was discernable. The down-regulation of miR-9-5p may result in higher *SIRT1* expression. The latter promoted the sprout behavior of EC and explained the neovascularization in diabetic retina. Furthermore, in tissue-specific expression data of ocular tissues in the Ocular Tissue Database (Wagner et al., 2013), we observed that *FOXO1* was highly expressed in normal choroid RPE, sclera, and retina, followed by *SIRT1* and *FOXO3* (Supplementary Figure 4). However, it is interesting to know whether or not this expression pattern changed under diabetic condition. Since the majority of the validated target genes were regulated by miR-9-5p, we think this miRNA would become a novel biomarker of a DR risk prediction and mechanism study.

Previous cell and animal models of DR have demonstrated the important roles of miRNAs in the pathology of DR. Most studies *in vitro* have investigated the roles of miRNAs in DR models of retinal endothelial cells (RECs) and retinal pigment epithelial (RPE) cells (Gong and Su, 2017). These models were based on the fact that REC and RPE are components of BRB, which were affected and impaired by the adverse effects of high glucose level under diabetes condition. On the other hand, many studies *in vivo* used streptozotocin (STZ)-induced diabetic animal models, which were possibly not the best model surrogating the etiology of retinopathy in T2DM patients.

Previous studies on the expression profile of DR miRNAs in human serum samples revealed several DR-specific miRNAs. For example, down-regulation of miR-17-3p was found in serum samples of Egyptian DR patients as compared with NDR patients (Shaker et al., 2019), which is consistent with our results (LFC = -3.64, *p*-value = 0.034). Another miRNA (miR-20b-3p) was not significant in our results. Especially in the Chinese population, we found case-control studies that could be closely related to our study in terms of the genetic background and environmental exposure (Zou et al., 2017; Liang et al., 2018; Liu et al., 2018). In general, the miRNAs reported in Chinese studies were not significant in our results (*p*-value > 0.05), but the directions of fold change were mostly consistent (**Table 3**). The reason behind the failure to replicate the previous reports may lie in the limited sample size, heterogeneity among statistical models and potential sampling bias.

**Table 3:** Significantly differentially expressed miRNAs in DR serum samples in Chinese T2DM patients in previous studies.

Authors	Year	Region	Methods	MiRNA	Reported FC	This study FC
Liang et al., 2018	2018	Shenzhen	RNA-seq and qRT-PCR	hsa-let-7a-5p	↓	↓
				hsa-miR-28-3p	↓	↓
				hsa-miR-151a-5p	↑	↓*
				hsa-miR-148a-3p	↑	↑
Liu et al., 2018	2018	Shenyang	qRT-PCR	hsa-miR-221	↑	↑ (hsa-miR-221-3p)
Zou et al., 2017	2017	Nanjing	qRT-PCR	hsa-miR-93	↑	↑ (hsa-miR-93-5p)


*↑ refers to up-regulated, ↓ refers to down-regulated. There was no statistical differences of these miRNAs in our study (p > 0.05). The latest mature miRNA names of the same mature form investigated in a previous study were provided in parentheses. The miRNA with inconsistent direction of fold change was highlight by asterisk. FC, fold change*.

## Conclusion

In conclusion, we unveiled novel DR-specific circulating miRNAs which may be functional in the early stage of DR. They could be employed both as biomarkers for early DR risk prediction and tools for exploring the molecular mechanism and therapeutic targets of DR. However, cautions need to be taken when interpreting these results since the limitations of current study warrants replication of these miRNAs in human serum samples or cell/animal models.

## Data Availability

The datasets generated for this study can be found in Sequence Read Archive, PRJNA540183.

## Ethics Statement

This study was carried out in accordance with the recommendations of China-Japan Union Hospital of Jilin University, with written informed consent from all subjects. All subjects gave written informed consent in accordance with the Declaration of Helsinki. The protocol was approved by the institutional review board of China-Japan Union Hospital of Jilin University.

## Author Contributions

ZL, YD, DL, and QW collected the blood samples and acquired the patient information. ZL, CH, XP, and PC conducted the analyses. ZL, CH, QW, and PC drafted the manuscript. XP, JY, LS, PC, and QW revised the manuscript and gave the final approval for submission.

## Conflict of Interest Statement

The authors declare that the research was conducted in the absence of any commercial or financial relationships that could be construed as a potential conflict of interest.
