# Supplementary material for: RNA-Seq Revealed Novel Non-proliferative Retinopathy Specific Circulating MiRNAs in T2DM Patients
Source: Front Genet. 2019 Jun 4;10:531. doi: 10.3389/fgene.2019.00531 (PMC6593299; doi:10.3389/fgene.2019.00531)
Supplement: Supplementary file 1 [file Data_Sheet_1.docx]

**RNA-seq Revealed Novel Non-proliferative Retinopathy Specific Circulating miRNAs in T2D Patients**

Zimeng Li^1^, Ying Dong^2^, Chang He^3,4^, Xingchen Pan^3,4^, Dianyuan Liu^1^, Jianli Yang^3^, Liankun Sun^5^, Peng Chen^3,6*†^, Qing Wang^1*†^

1 Department of Endocrinology, China-Japan Union Hospital, Jilin University, Changchun, China.

2 Department of Radiotherapy, The Tumor Hospital of Jilin Province, Changchun, China.

3 Department of Genetics, College of Basic Medical Sciences, Jilin University, Changchun, China.

4 Department of Molecular Biology, College of Basic Medical Sciences, Jilin University, Changchun, China.

5 Department of Pathophysiology, College of Basic Medical Sciences, Jilin University, Changchun, China.

6 Department of Pathology, College of Basic Medical Sciences, Jilin University, Changchun, China.

† These authors contributed equally to this work.

* Correspondence:

Peng Chen

[pchen@jlu.edu.cn](mailto:pchen@jlu.edu.cn)

Qing Wang

[wang_qing@jlu.edu.cn](mailto:wang_qing@jlu.edu.cn)

**Supplementary Figures**

Supplementary Figure 1. ROC curve of individual miRNAs. The ROC analysis was done for each differentially expressed miRNA, labeled on top of each subfigure. In each subfigure, X-axis represents the specificity, while Y-axis represent the sensitivity. The shade area is the estimated 95% confidence interval.


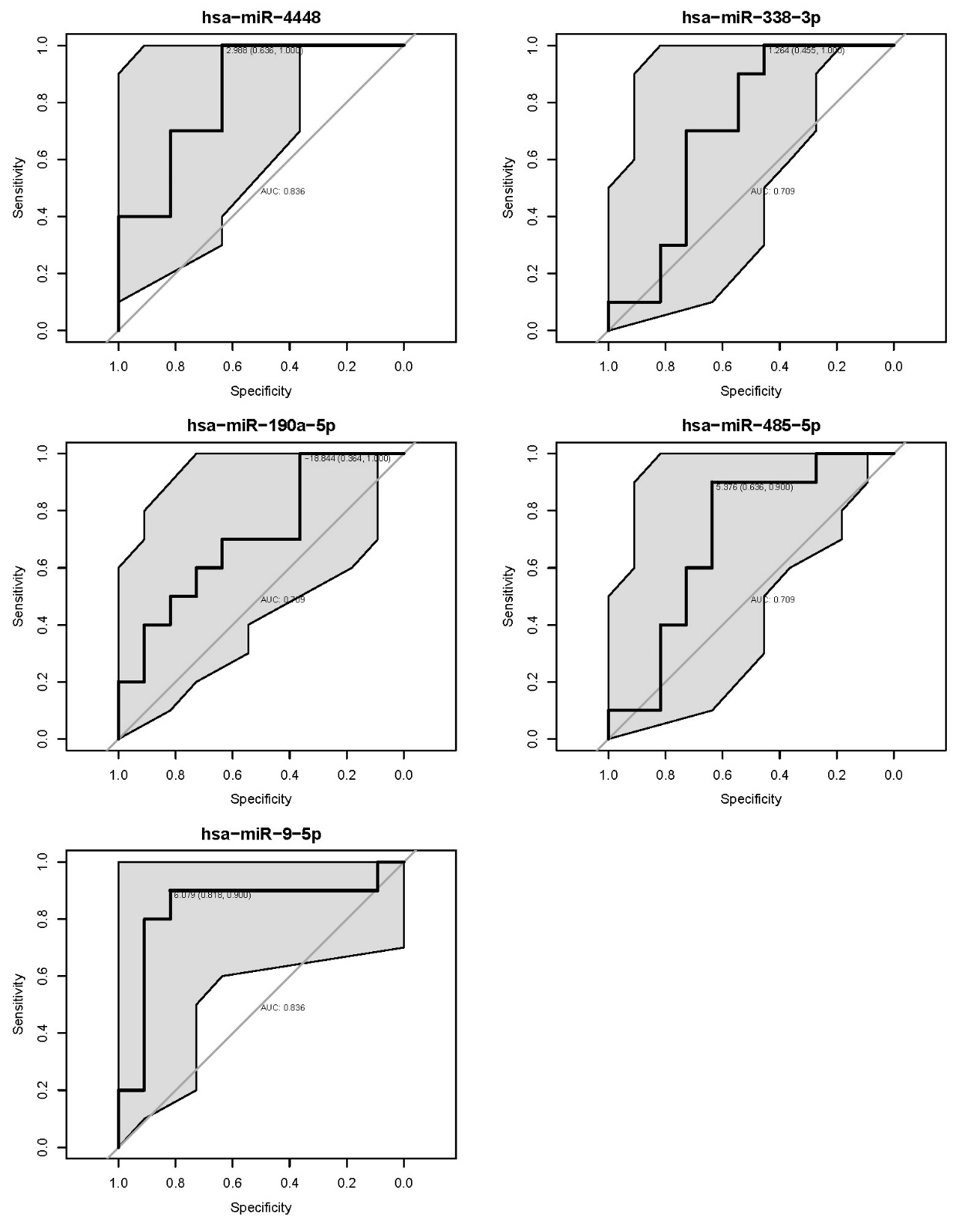


Supplementary Figure 2. Polygenic risk scores in DR (case) and NDR (control) groups. This is the boxplot of the calculated polygenic risk score using selected miRNAs (shown in the main text). The plot was divided by two groups, DR and NDR. Y-axis represent the calculated score, while *t*-test p value is stated between the two boxes.


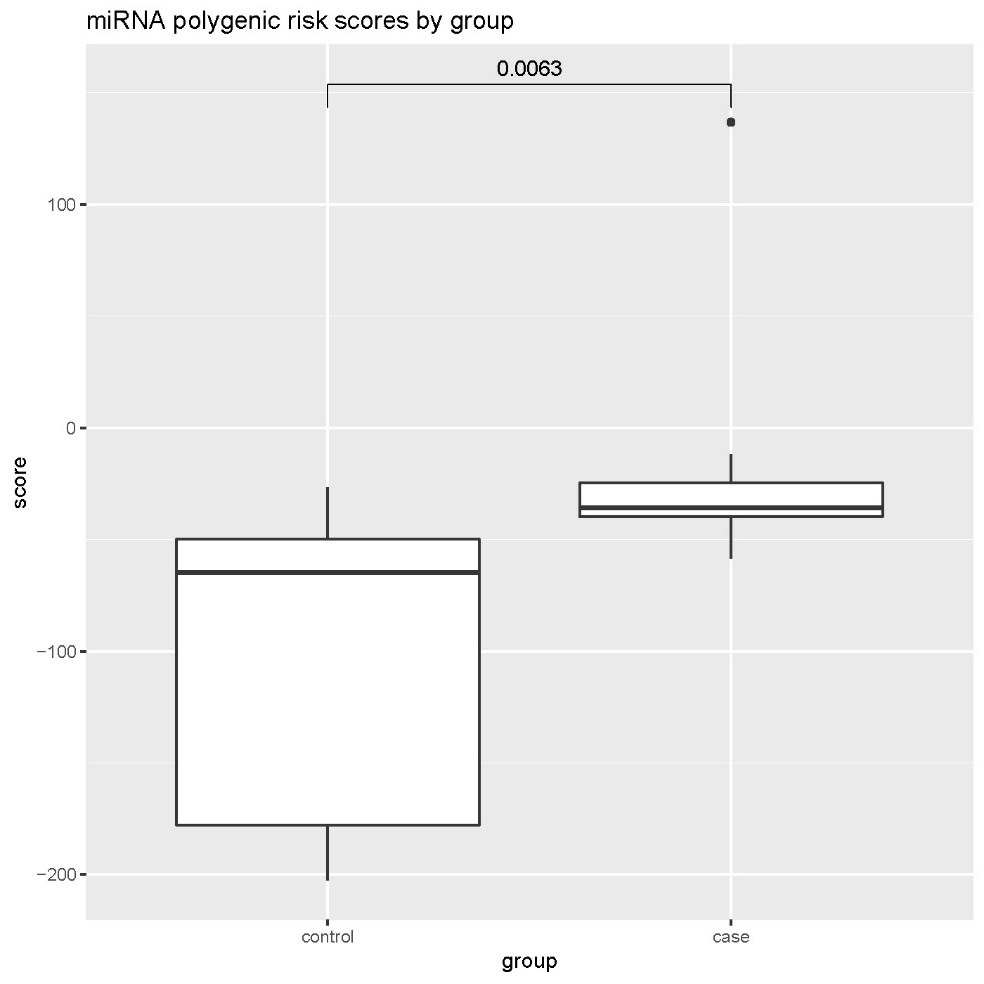


Supplementary Figure 3. The illustration of NAD metabolism, sirtuins and aging pathway. This figure originated from <https://www.wikipathways.org/index.php/Pathway:WP3630>.


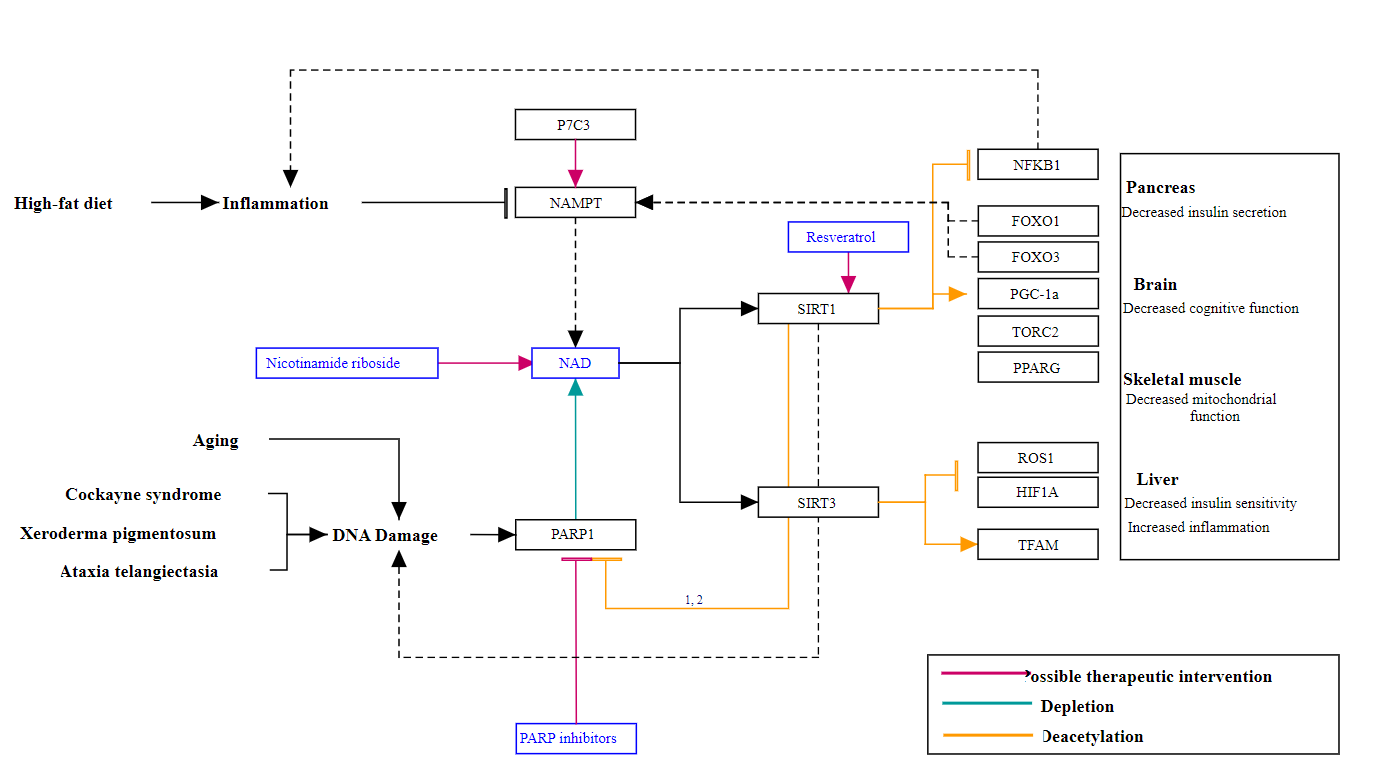


Supplementary Figure 4. Tissue-specific expression of miR-9-5p target genes in sirtuin pathway. Y-axis represents the PLIER value (Probe Logarithmic Intensity ERror), a measurement of the probe signal strength used in Affymetrix arrays (PMID: 19259420).


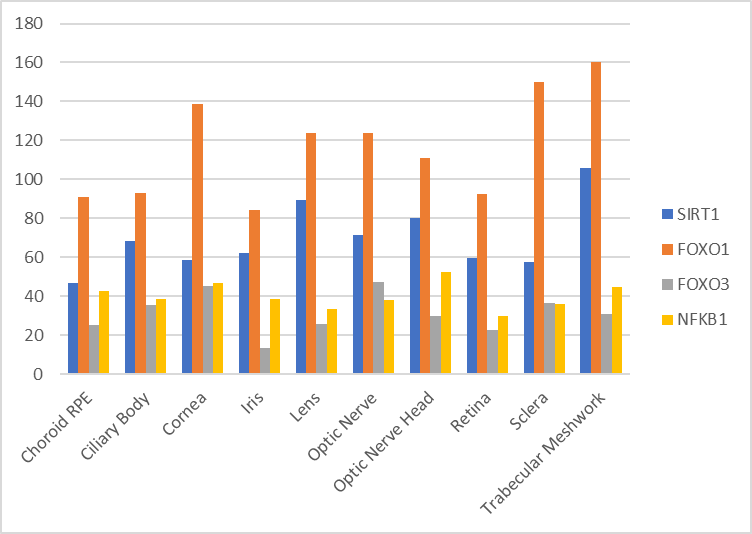


**Supplementary Tables**

Supplementary Table 1. Target genes of DE miRNAs.

| Mature miRNA | Target gene symbol |
| --- | --- |
| miR-4448* | *ADAMTS6, ARID2, AUTS2, BPTF, BRWD3, CCDC88A, CHD2, FAM117B, FAM177A1, GRID2, HOXB6, KATNBL1, LRRN1, MAT1A, NEURL4, OPHN1, PDE8B, PRKAR2A, RGS4, SRGAP2, TBL1XR1, TMEM132C, ZNF436, ZNFX1* |
| miR-338-3p | *CCND1, MACC1, NRP1, PLA2G4B, PREX2, SMO, SSX2IP, ZEB2* |
| miR-190a-5p | *CDKN1B, IGF1, KCNQ5, MARK2, PHLPP1* |
| miR-485-5p | *APOA5, HIF3A* |
| miR-9-5p | *ACAT1, AP3B1, BACE1, BCL2L11, BCL6, CCND1, CCNG1, CDX2, CREB1, CXCR4, DICER1, DRD2, ELAVL1, ETS1, FOXO1, FOXO3, FOXP1, HDAC4, ID2, KLF17, MALAT1, MIRLET7D, MMP13, MTHFD1, NF1, NFKB1, NR2E1, NTRK3, ONECUT2, POU2F2, PPARA, PRDM1, PRTG, RAB34, REST, SIRT1, SOCS5, SRF, STMN1, TGFBI, TGFBR2* |

*, no experiment validated target genes available, instead, predicted target genes were presented.
